# Overestimation of Aortic Stenosis in Thiamine Deficiency: Two Cases With Distinct Underlying Conditions

**DOI:** 10.7759/cureus.99487

**Published:** 2025-12-17

**Authors:** Satoshi Kurisu, Hitoshi Fujiwara

**Affiliations:** 1 Department of Cardiology, National Hospital Organization (NHO) Hiroshima-Nishi Medical Center, Otake, JPN

**Keywords:** beriberi, echocardiography, high-output, valvular heart disease, vitamin

## Abstract

The severity of aortic stenosis (AS) can be overestimated in high-flow states, and thiamine deficiency is an underrecognized cause of high-output circulatory states. We report two elderly females who developed thiamine deficiency-related high-output circulatory states, initially leading to an overestimation of AS severity. Case 1 was assessed as severe but was downgraded to moderate after thiamine replacement, and case 2 was initially assessed as moderate but was reclassified as mild after treatment. Case 1 had a history of gastrectomy, predisposing her to thiamine deficiency due to limited postoperative intake and a more alkaline gastric environment. Case 2 had liver cirrhosis and was on long-term spironolactone and loop diuretics, contributing to thiamine depletion via renal losses and impaired gastrointestinal utilization. Both patients exhibited refractory edema and hyperdynamic left ventricular wall motion. Thiamine deficiency impairs adenosine triphosphate production, reducing systemic vascular resistance and triggering compensatory high-output circulatory states. Prompt thiamine replacement improved symptoms and hemodynamic parameters. These cases highlight the importance of recognizing reversible high-flow states to avoid misinterpretation of AS severity and ensure accurate evaluation and management.

## Introduction

Thiamine (vitamin B1) deficiency, an underrecognized cause of high-output circulatory states, can result from conditions that impair nutrient absorption, such as gastrointestinal surgery [1], as well as from prolonged diuretic therapy [2-4], malnutrition [1], chronic alcoholism [5], dialysis [6], and states associated with increased metabolic demand, such as thyrotoxicosis [7]. It may present with refractory edema and induce a high-output circulatory state, commonly referred to as wet beriberi [8,9]. Such a high-output state can alter echocardiographic parameters and potentially lead to misinterpretation of valvular heart disease severity [10].

Here, we present two elderly females who developed thiamine deficiency-related high-output circulatory states, resulting in hyperdynamic left ventricular (LV) wall motion, attributable to different underlying conditions: one after gastrectomy and the other with liver cirrhosis on long-term diuretic therapy. In both cases, echocardiographic findings initially overestimated the severity of aortic stenosis (AS) due to thiamine deficiency.

## Case presentation

Case 1

An 88-year-old female with hypertension, diabetes mellitus, and hypothyroidism, with a history of gastrectomy for gastric cancer, presented with a two-week history of refractory leg edema. She was taking oral azosemide (30 mg/day) for edema and several antihypertensive agents, including carvedilol, azilsartan, and benidipine. She was also taking levothyroxine. Physical examination revealed a grade 3/6 systolic murmur and bilateral pitting edema. Laboratory tests showed anemia and hypoalbuminemia (Table 1).

**Table 1:** Laboratory data in case 1.

Variable	Initial presentation	Follow-up	Reference range
Blood count			
White blood cell count (/µL)	3.6 × 10^3^	3.0 × 10^3^	3.3 - 8.6 × 10^3^
Red blood cell count (/µL)	2.63 × 10^6^	3.20 × 10^6^	4.86 - 4.92 × 10^6^
Hemoglobin (g/dL)	8.8	10.4	11.6 - 14.8
Platelet count (/µL)	195 × 10^3^	212 × 10^3^	158 - 348 × 10^3^
Blood chemistry			
Aspartate aminotransferase (U/L)	31	24	13 - 30
Alanine aminotransferase (U/L)	19	9	7 - 23
Lactate dehydrogenase (U/L)	252	257	124 - 222
Total protein (g/dL)	6.2	6.2	6.6 - 8.1
Albumin (g/dL)	3.3	2.7	4.1 - 5.1
Blood urea nitrogen (mg/dL)	22.7	31.6	8 - 20
Creatinine (mg/dL)	1.09	1.15	0.46 - 0.79
Sodium (mmol/L)	144	143	138 - 145
Potassium (mmol/L)	3.9	3.7	3.6 - 4.8
Chloride (mmol/L)	111	103	101 - 108
Glucose (mg/dL)	92	-	73 - 109
Thyroid-stimulating hormone (µIU/mL)	19.58	-	0.61 - 4.23
Free triiodothyronine (pg/mL)	1.90	-	1.68 - 3.67
Free thyroxine (ng/dL)	0.51	-	0.7 - 1.48
Thiamine (vitamin B1) (ng/mL)	15	173	24 - 66
Vitamin B12 (pg/mL)	525	-	187 - 883

**Figure 1:**
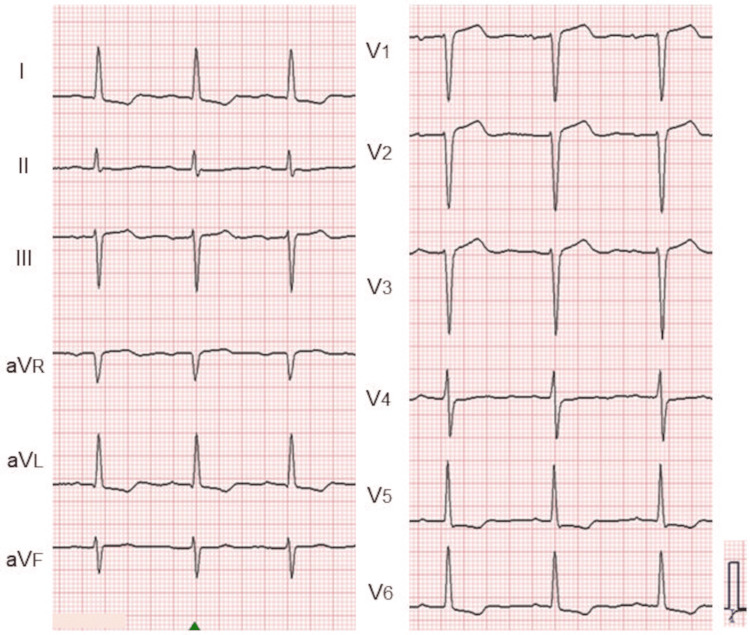
Electrocardiogram in case 1. Electrocardiography demonstrated a normal sinus rhythm with a left ventricular strain pattern.

**Figure 2:**
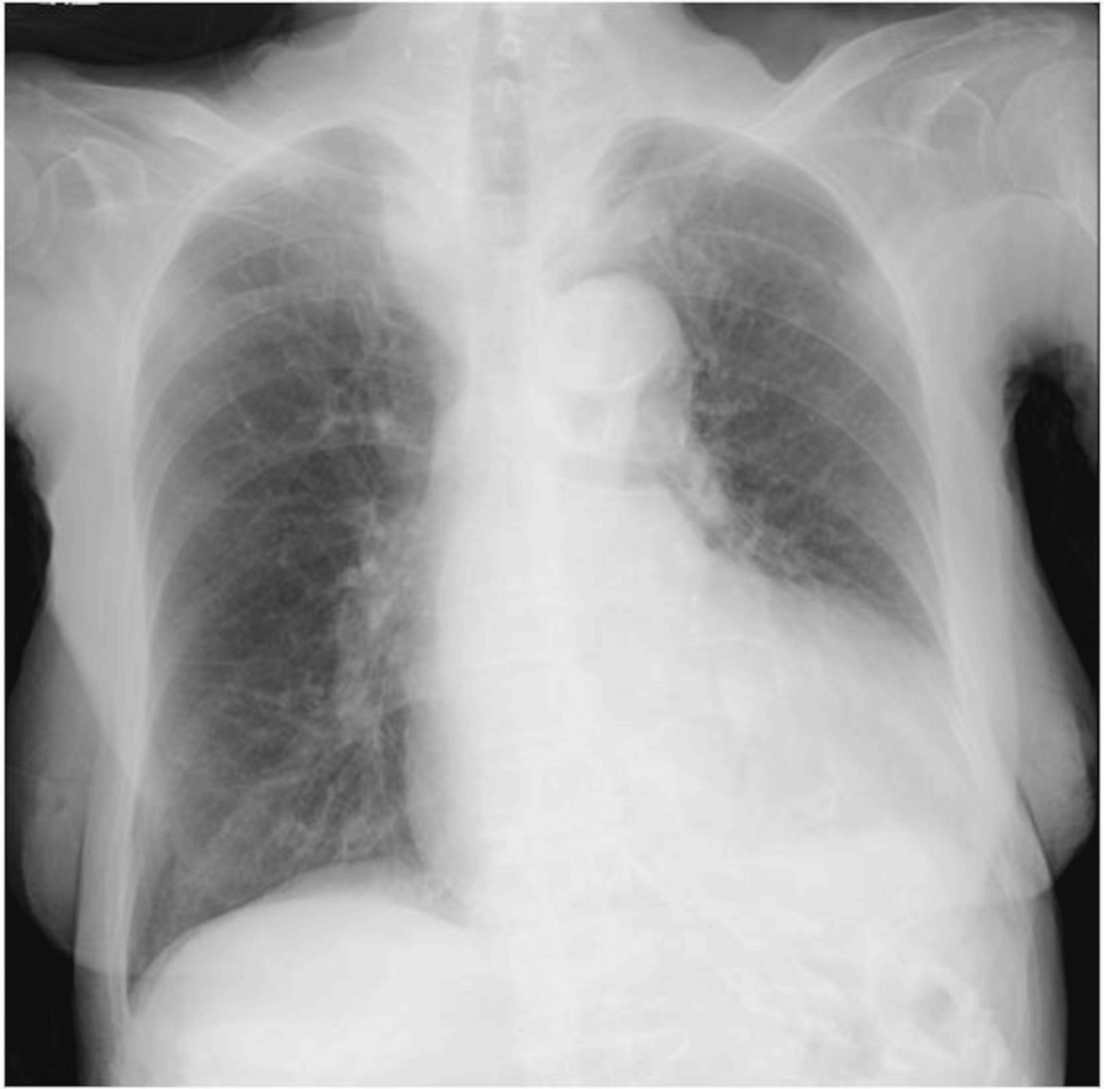
Chest radiograph in case 1. Chest radiography revealed cardiomegaly.

Electrocardiography demonstrated a normal sinus rhythm with an LV strain pattern (Figure 1), and chest radiography revealed cardiomegaly (Figure 2).

**Figure 3:**
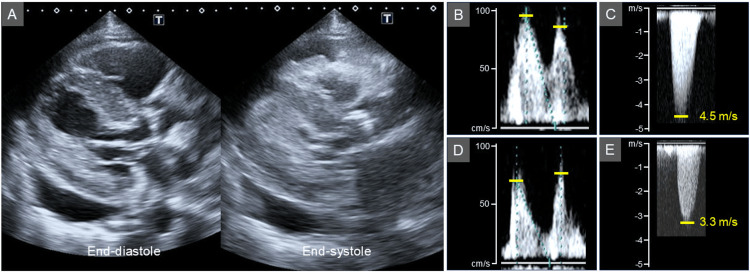
Transthoracic echocardiographic images in case 1. Transthoracic echocardiography showed a high ejection fraction (EF) of 80% (A), left ventricular hypertrophy, a calcified aortic valve, an early to late diastolic filling velocity ratio (E/A) of 1.2 (B), and pericardial effusion. The peak aortic jet velocity was 4.5 m/s (C). After thiamine supplementation, EF decreased to 50%, with a change in the E/A to 0.9 (D), and a reduction in peak aortic jet velocity to 3.3 m/s (E).

Transthoracic echocardiography showed a high ejection fraction (EF) of 80% (Figure 3A), LV hypertrophy, a calcified aortic valve, an early to late diastolic filling velocity ratio (E/A) of 1.2 (Figure 3B), and pericardial effusion. The LV outflow tract-velocity time integral (LVOT-VTI) was 19.7 cm, a parameter related to stroke volume. The peak aortic jet velocity was 4.5 m/s (Figure 3C) and the mean transvalvular pressure gradient was 46 mmHg, both consistent with severe AS (Table 2).

**Table 2:** Transthoracic echocardiographic parameters in case 1. LVDD, left ventricular end-diastolic dimension; LVEF, left ventricular ejection fraction; E/A, early to late diastolic filling velocity ratio; LVOT-VTI, left ventricular outflow tract velocity time integral.

	Initial presentation	Follow-up
LVDD (mm)	44	40
LVEF (%)	80	50
E/A	1.2	0.9
LVOT-VTI (cm)	19.7	13.1
Peak aortic jet velocity (m/s)	4.5	3.3
Mean pressure gradient (mmHg)	46	24

Given the hyperdynamic LV wall motion and her history of gastrectomy, thiamine deficiency was suspected, and intravenous fursultiamine was empirically administered after blood sampling, followed by oral replacement (75 mg/day). The pretreatment thiamine level was 15 ng/mL (reference range, 24-66 ng/mL), confirming the diagnosis.

One month after starting thiamine replacement, the thiamine level had increased to 28 ng/mL, and her edema had improved. After eight months, the thiamine level further increased to 173 ng/mL (Table 1), accompanied by a decrease in EF to 50%, a change in the E/A to 0.9 (Figure 3D), a reduction in LVOT-VTI to 13.1 cm, and a decrease in the peak aortic jet velocity to 3.3 m/s (Figure 3E). Pericardial effusion was reduced but remained mild. Upon reassessment after thiamine replacement, the true severity of AS was considered moderate. The patient has remained clinically stable under conservative management with continued thiamine replacement over 14 months of follow-up.

Case 2

An 89-year-old female with liver cirrhosis on long-term diuretic therapy presented with an eight-week history of refractory leg edema and a three-week history of a non-healing right lower leg ulcer following a skin injury. Her medications included azosemide (30 mg/day), furosemide (10 mg/day), and spironolactone (25 mg/day), along with branched-chain amino acids. She had hypothyroidism and was receiving levothyroxine. She had no history of gastrointestinal surgery. On physical examination, a grade 3/6 systolic murmur and bilateral pitting edema were noted. Laboratory tests showed anemia and hypoalbuminemia (Table 3).

**Table 3:** Laboratory data in case 2.

Variable	Initial presentation	Follow-up	Reference range
Blood count			
White blood cell count (/µL)	3.7 × 10^3^	4.5 × 10^3^	3.3 - 8.6 × 10^3^
Red blood cell count (/µL)	3.46 × 10^6^	3.61× 10^6^	4.86 - 4.92 × 10^6^
Hemoglobin (g/dL)	7.2	7.4	11.6 - 14.8
Platelet count (/µL)	229 × 10^3^	217 × 10^3^	158 - 348 × 10^3^
Blood chemistry			
Aspartate aminotransferase (U/L)	29	33	13 - 30
Alanine aminotransferase (U/L)	14	17	7 - 23
Lactate dehydrogenase (U/L)	258	261	124 - 222
Total protein (g/dL)	6.8	7.2	6.6 - 8.1
Albumin (g/dL)	3.0	3.1	4.1 - 5.1
Blood urea nitrogen (mg/dL)	27.7	35.3	8 - 20
Creatinine (mg/dL)	1.05	1.01	0.46 - 0.79
Sodium (mmol/L)	138	137	138 - 145
Potassium (mmol/L)	3.9	4.1	3.6 - 4.8
Chloride (mmol/L)	105	104	101 - 108
Glucose (mg/dL)	92	-	73 - 109
C-reactive protein (mg/dL)	0.07	-	0 - 0.14
Thiamine (vitamin B1) (ng/mL)	20	237	24 - 66

**Figure 4:**
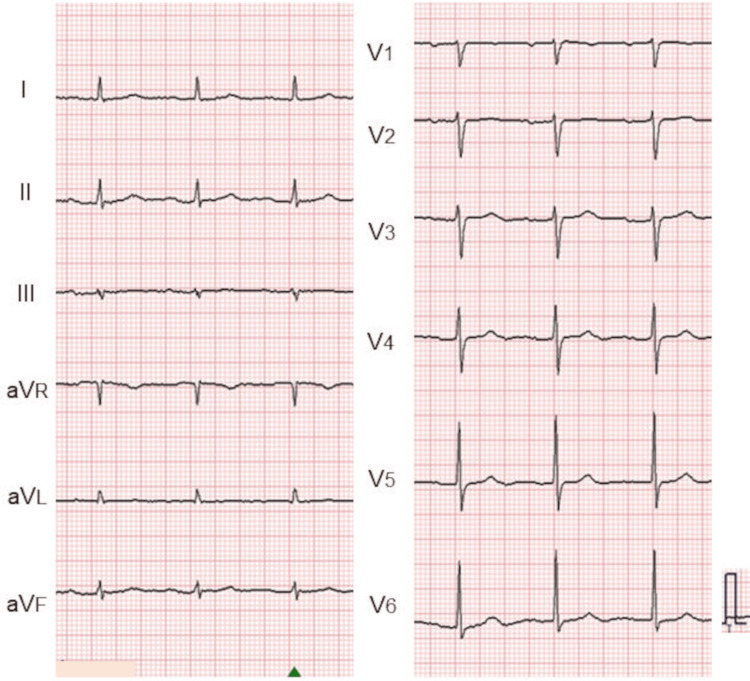
Electrocardiogram in case 2. Electrocardiography demonstrated normal sinus rhythm.

**Figure 5:**
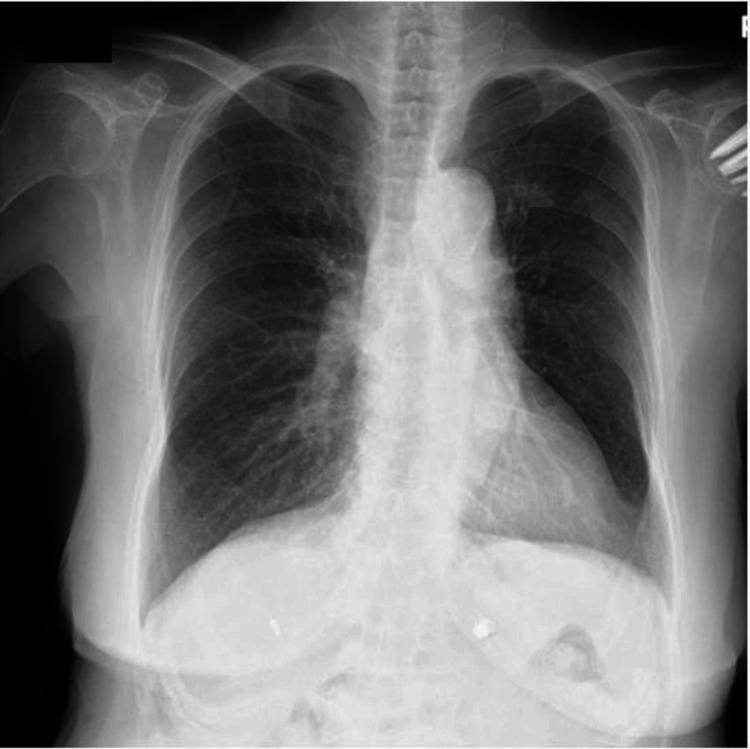
Chest radiograph in case 2. Chest radiography showed a cardiothoracic ratio of 52%.

Electrocardiography demonstrated a normal sinus rhythm (Figure 4), and chest radiography showed a cardiothoracic ratio of 52% (Figure 5).

**Figure 6:**
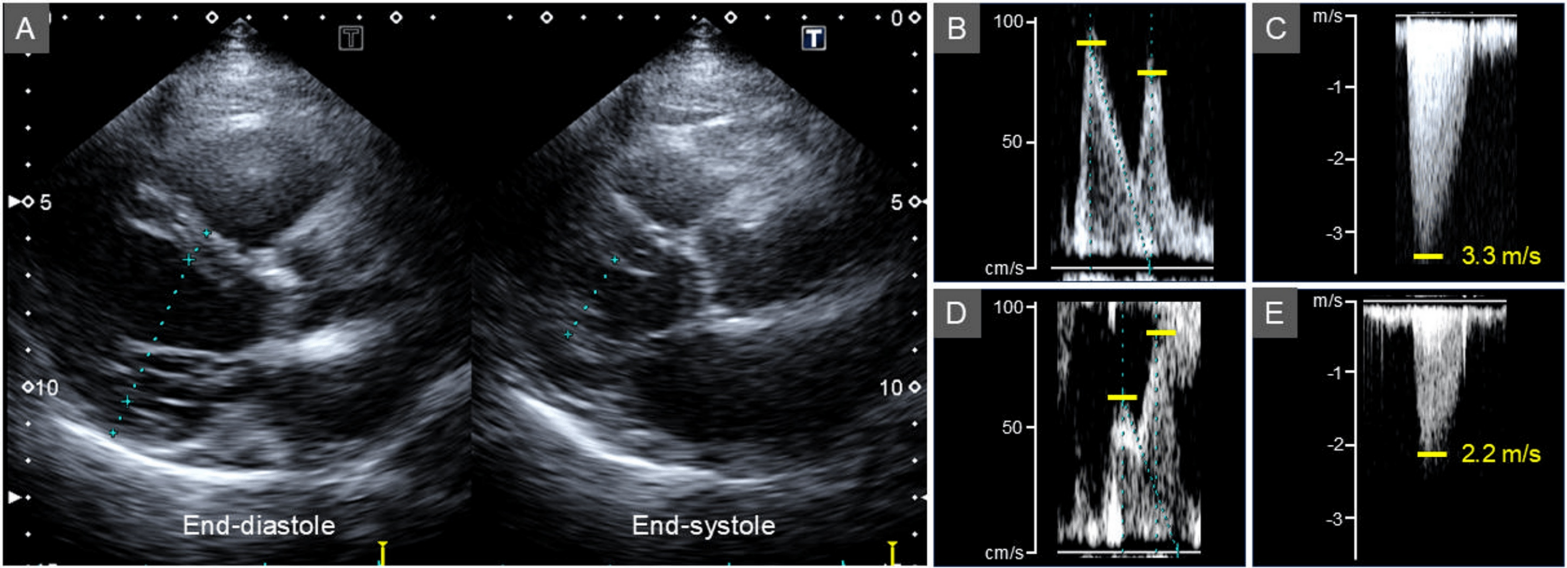
Transthoracic echocardiographic images in case 2. Transthoracic echocardiography revealed a high ejection fraction (EF) of 74% without left ventricular hypertrophy (A), a calcified aortic valve, an early to late diastolic filling velocity ratio (E/A) of 1.2 (B), and a peak aortic jet velocity of 3.3 m/s (C). After thiamine supplementation, EF decreased to 68%, with a change in the E/A to 0.7 (D), and the peak aortic jet velocity declined to 2.2 m/s (E).

Transthoracic echocardiography revealed a high EF of 74% without LV hypertrophy (Figure 6A), a calcified aortic valve, an E/A of 1.2 (Figure 6B), and an LVOT-VTI of 26.6 cm. The peak aortic jet velocity of 3.3 m/s (Figure 6C) and the mean transvalvular pressure gradient of 20 mmHg were consistent with moderate AS (Table 4).

**Table 4:** Transthoracic echocardiographic parameters in case 2. LVDD, left ventricular end-diastolic dimension; LVEF, left ventricular ejection fraction; E/A, early to late diastolic filling velocity ratio; LVOT-VTI, left ventricular outflow tract velocity time integral.

	Initial presentation	Follow-up
LVDD (mm)	42	40
LVEF (%)	74	68
E/A	1.2	0.7
LVOT-VTI (cm)	26.6	19.7
Peak aortic jet velocity (m/s)	3.3	2.2
Mean pressure gradient (mmHg)	20	14

Because of the hyperdynamic LV wall motion and persistent edema despite diuretic therapy, thiamine deficiency was suspected. Oral fursultiamine (75 mg/day) was started empirically after blood sampling, and the diagnosis was subsequently supported by a low pretreatment thiamine level (20 ng/mL). Within one month, partial improvement of the edema and leg ulcer was observed. After three months, both had resolved completely, accompanied by an increase in thiamine level to 237 ng/mL. EF decreased to 68%, E/A changed to 0.7 (Figure 6D), and LVOT-VTI decreased to 19.7 cm, with a corresponding decline in the peak aortic jet velocity to 2.2 m/s (Figure 6E), indicating hemodynamic stabilization. The true severity of AS was reassessed as mild. The patient remained clinically stable under conservative management with continued thiamine replacement over a six-month follow-up period.

## Discussion

In this report, we describe two elderly females who developed thiamine deficiency-related high-output circulatory states with hyperdynamic LV wall motion. One patient had a history of gastrectomy, while the other had liver cirrhosis and was on long-term diuretic therapy. In both cases, the severity of AS was initially overestimated.

As demonstrated in case 1, gastrectomy can predispose patients to thiamine deficiency, not only because postoperative nutritional intake is often limited, but also because the rise in gastric pH after surgery creates a more alkaline environment that renders thiamine unstable and reduces its availability [11,12]. In case 2, liver cirrhosis was associated with persistent fluid retention, and long-term therapy with spironolactone and loop diuretics likely contributed to thiamine depletion via increased renal losses and reduced gastrointestinal absorption [2-4]. In both cases, refractory edema developed before presentation, and initial echocardiography revealed hyperdynamic LV wall motion, raising suspicion for thiamine deficiency.

Thiamine deficiency impairs adenosine triphosphate production, leading to adenosine accumulation. This, in turn, reduces systemic vascular resistance, triggering a compensatory high-output circulatory state with elevated blood volume [13]. Long-term diuretic therapy, while effective in controlling fluid retention, can lead to increased urinary loss of water-soluble vitamins, including thiamine [2-4]. Clinicians need to be aware that diuretic therapy, though intended to alleviate edema, may paradoxically lead to thiamine deficiency and thereby exacerbate edema.

In addition to thiamine deficiency, conditions such as liver cirrhosis, anemia, thyrotoxicosis, sepsis, and arteriovenous shunts may also cause a high-output circulatory state [14,15]. In our report, both case 1 and case 2 had anemia, which had been managed at another institution. Therefore, no detailed evaluation or intervention for anemia was undertaken at our hospital. Case 2 also had underlying liver cirrhosis, which was not amenable to specific intervention. Despite the presence of these potential contributors, both patients showed prompt symptomatic and hemodynamic improvement after thiamine replacement, suggesting that thiamine deficiency played a major role in the observed high-output states. Importantly, in these cases, the high-output state caused by thiamine deficiency initially led to an overestimation of AS severity.

Peak aortic jet velocity is highly flow-dependent, which is a major limitation. This can lead to substantial overestimation of AS severity in high-flow states, such as concomitant aortic regurgitation, anemia, thyrotoxicosis, or thiamine deficiency [10], and to underestimation of AS severity in low-flow conditions [16]. When a high-output state is suspected, particularly when peak velocity or transvalvular gradients appear disproportionately elevated relative to the clinical presentation, it is recommended to first identify and appropriately manage reversible causes of increased flow, followed by reassessment once the patient’s hemodynamics have stabilized [17]. Reassessment after normalization of flow allows echocardiographic findings to be incorporated more appropriately into final clinical decision-making, including whether conservative management or valve intervention is warranted. In line with this recommendation, the two cases presented here, both complicated by thiamine deficiency-induced high-output states, highlight the importance of correcting reversible high-flow conditions to allow an accurate evaluation of AS severity. Regarding thiamine deficiency, rather than advocating routine screening, our findings suggest that targeted evaluation should be considered in selected patients with high-flow states and risk factors for deficiency, such as malnutrition, chronic diuretic use, a history of gastrectomy, alcoholism, or prolonged inadequate oral intake.

An important limitation of this report is that biomarkers of volume status, such as natriuretic peptide levels, and invasive hemodynamic assessment by right heart catheterization were not obtained. In addition, the follow-up period was limited, and the time course to major improvement differed between the two cases, approximately eight months in case 1 and three months in case 2, making it difficult to determine whether these recovery intervals represent typical timelines or reflect patient-specific factors.

## Conclusions

In conclusion, these cases illustrate that reversible high-output states can substantially influence the echocardiographic assessment of AS severity. In patients with high-flow states, identification and correction of possible causes of increased flow, including thiamine deficiency, followed by reassessment after hemodynamic stabilization, are essential to ensure accurate evaluation and appropriate clinical decision-making, including whether conservative management or valve intervention is warranted.
